# Correlations between FTO Gene Polymorphisms and TSH Level in Uyghur Chinese Patients with Type 2 Diabetes

**DOI:** 10.1155/2021/6646750

**Published:** 2021-06-26

**Authors:** Li Wang, QiZhong Yi, Hua Yao, LiJuan He, BinBin Fang, WeiFang Xu, YinXia Su, JiWen Liu, Qi Ma

**Affiliations:** ^1^Key Laboratory of Xinjiang Metabolic Disease, Clinical Medical Research Institute, The First Affiliated Hospital of Xinjiang Medical University, Urumqi 830054, China; ^2^Departments of Occupational and Environmental Health, Xinjiang Medical University, Urumqi 830054, China; ^3^Psychological Medicine Center, The First Affiliated Hospital of Xinjiang Medical University, Urumqi 830054, China; ^4^Clinical Medical Research Institute, The First Affiliated Hospital of Xinjiang Medical University, Urumqi 830054, China; ^5^Anesthesiology, The First Affiliated Hospital of Xinjiang Medical University, Urumqi 830054, China; ^6^Health Management Centre, The First Affiliated Hospital of Xinjiang Medical University, Urumqi 830054, China

## Abstract

**Objective:**

The aim of this study was to investigate the correlation between polymorphisms in the FTO gene and TSH level in Uyghur patients with type 2 diabetes in the Xinjiang region. *Material and Methods*. This cohort was made up of 498 Uyghur patients with type 2 diabetes who underwent genotype screening for rs8050136 and rs9939609 using the Sequenom MassARRAY system. The distribution frequencies of the genotypes and alleles at rs8050136 and rs993960 were compared between two patient groups, those with TSH < 2.5 mU/L and those with TSH ≥ 2.5 mU/L group. We further evaluated the relationships between these different genotypes and FT3, FT4, TSH, FPG, and HbA1c expression.

**Results:**

The results suggested the TSH level was 2.281 times higher in rs8050136 CC+CA carriers than in AA genotype (95%CI = 1.024~5.080, *P* = 0.044) and was 2.417 times higher in rs9939609 TT+TA carriers than in AA genotype (95%CI = 1.257~4.649, *P* = 0.008) after adjusting for age, sex, and BMI under the recessive model. TSH levels were significantly different between T2DM patients with different FTO genotypes, rs8050136 (*P* = 0.008) and rs9939609 (*P* = 0.003), with TSH levels in rs8050136 CC genotype carriers showing a significant increase compared to those in the AA genotype carriers (*P* = 0.005). Additionally, rs9939609 TT and TA genotype carriers had a significant increase in the TSH level when compared to AA genotype carriers (*P* = 0.001 and *P* = 0.031, respectively). The TSH level was also significantly different in these male patients with different genotypes of rs8050136 (*P* = 0.026) and rs9939609 (*P* = 0.019). And TSH levels in rs8050136 CC genotype male carriers showing a significant increase compared to those in the AA genotype carriers (*P* = 0.013) and rs9939609 TT genotype male carriers had a significant increase in TSH level when compared to AA genotype carriers (*P* = 0.004).

**Conclusion:**

The polymorphisms at rs8050136 and rs9939609 are associated with changes in the TSH level with rs8050136 CC and rs9939609 TT genotypes identified as potential risk factors for increased TSH levels in these male patients.

## 1. Introduction

There are an estimated 463 million patients with diabetes across the world, and this number continues to increase every year. China, which has 116 million patients with diabetes, has the largest population of diabetics in the world [1]. Diabetes can account for up to 20 percent of the national health budget. There are 4.2 million diabetes-associated deaths every year which majorly affect health and socioeconomic conditions of the population. The number of patients with thyroid dysfunction in diabetic patients was significantly higher than that in non-diabetic patients.

There is a common etiological basis for diabetes mellitus and thyroid disease which may be based on genetic defects or susceptibility. A meta-analysis of the global data suggests that about 11% of the patients with diabetes also exhibit some form of thyroid dysfunction [2]. This thyroid dysfunction may be related to genetics, but the mechanism remains unclear.

The fat mass and obesity associated (FTO) gene contains 9 exons and 8 introns, expresses a 410.50 kbp full-length construct, and is located on human chromosome 16. The FTO gene which plays an important role in controlling energy balance has been closely linked to obesity. Many diseases, including obesity [3], T2DM [4], and cancer, have been reported to be associated with the FTO gene. A study has shown [5] that FTO expression correlates with thyroid stimulating hormone (TSH) regulation in overweight/obese children. It suggested that FTO may be a common genetic susceptibility gene for diabetes and thyroid dysfunction which caused the two diseases to overlap. However, there are relatively few reports that discuss the correlation between FTO gene polymorphisms and thyroid hormone abnormalities in T2DM. Therefore, it is of vital importance to early diagnose susceptible genes of thyroid dysfunction in T2DM for diabetic control. In this study, we evaluated the relationship between FTO gene polymorphisms and TSH level in Uyghur patients with diabetes in Xinjiang.

## 2. Materials and Methods

### 2.1. Study Population

All 498 Uyghur patients presenting with T2DM were recruited for this study from the Xinjiang Uyghur Autonomous Region of China. This cohort included 324 males and 174 females, aged from 20 to 80 years, with an average age of 51.22 ± 9.45 years. The study subjects were diagnosed with T2DM at the First Affiliated Hospital of Xinjiang Medical University between October 2016 and February 2017. A total of 498 cases of T2DM were diagnosed using the diagnostic criteria for T2DM described by the World Health Organization (WHO) 1999 (classic diabetes symptoms plus any of the following: (1) fasting blood glucose ≥ 7.0 mmol/L; (2) random blood glucose ≥ 11.1 mmol/L; and (3) oral glucose tolerance test (OGTT) for 2 h ≥ 11.0 mmol/L). Participants with type I diabetes, maturity onset diabetes of the young (MODY), gestational diabetes, stress-induced hyperglycemia, malignant tumors, chronic infections, neuropsychiatric diseases, autoimmune diseases, heart disease, or liver disease were excluded. This study was approved by the Ethics Committee of the First Affiliated Hospital of Xinjiang Medical University.

Participants were interviewed using a structured questionnaire by trained personnel to collect demographic data (age, gender, and so on). Body weight, height, and blood pressure were measured using standard procedures, and body mass index (BMI) was calculated as the subject's weight (kg) divided by the square of the subject's height (m^2^). For the clinical chemistry assays, peripheral venous blood samples were obtained from each participant after a 12-hour fast, and the serum was separated and stored at −70°C. These serum samples were then used for various biochemical assays including FBG, TSH, free triiodothyronine (FT3), free theroxine (FT4), total cholesterol (TC), triacylglycerol (TG), high-density lipoprotein cholesterol (HDL-C), and low-density lipoprotein cholesterol (LDL-C) in accordance with the procedures recommended by the Clinical Laboratory Department of the First Affiliated Hospital of Xinjiang Medical University.

### 2.2. Single-Nucleotide Polymorphism Genotyping

Genomic DNA was isolated from peripheral blood leukocytes using the Wizard Genomic DNA Purification kit (Promega Corp., Madison, WI, USA) according to the manufacturer's instructions. The SNPs were genotyped using the Sequenom MassARRAY system (San Diego, CA, USA) according to the iPLEX Gold Application Guide. The sequences of the primers for FTO rs8050136 were as follows: F—ACGTTGGATGCCATGAGTCCATCTCTACAG, R—ACGTTGGATGTAGTCTAGGCATGCCAGTTG, and rs9939609 were as follows: F—GCGGTCCCAAAAGGGTCAGTCTTGCGACTGCTGTGAATCT, R—GCGGTCCCAAAAGGGTCAGTGTTAATGGCTTCAGGGTACCAG. These SNP evaluations passed the relevant quality control criteria with a genotyping call rate of >90%.

### 2.3. Statistical Analyses

All statistical analyses were performed using the Statistical Package for Social Sciences version 22.0 (SPSS Inc., Chicago, IL, USA). Continuous variables are expressed as the mean ± standard deviation and were analyzed by one-way ANOVA. The chi-square test was used to compare the rate of the genotype/allele of each SNP among the study participants. The Hardy–Weinberg equilibrium (HWE) was evaluated using the homogeneity *χ*^2^ test. Logistic regression analysis was carried out to evaluate the relationship between each factor and TSH level. *P* values of <0.05 were considered statistically significant.

## 3. Results

### 3.1. Comparison of the General Clinical Characteristics of T2DM Patients with Different TSH Levels

The TSH level in all 498 subjects was bounded at 2.5 mU/L; subjects with a TSH < 2.5 mU/L were included in the TSH-0 group, and those with a TSH ≥ 2.5 mU/L were placed in the TSH-1 group. There were statistically significant differences in gender and BMI between the two groups (*P* < 0.05). However, there were no statistically significant differences in age, SBP, DBP, FPG, HbA1c, FT3, FT4, TC, TG, HDL-C, and LDL-C (*P* > 0.05). The comparisons of the general clinical data for this cohort are summarized in Table 1.

Continuous variables are expressed as the mean ± standard deviation. BMI: body mass index; SBP: systolic blood pressure; DBP: diastolic blood pressure; FPG: fasting plasma glucose; HbA1c: hemoglobin A1c; FT3: free triiodothyronine; FT4: free theroxine; TC: serum total cholesterol; TG: triglyceride; HDL-C: high-density lipoprotein cholesterol; LDL-C: low-density lipoprotein cholesterol.

### 3.2. Comparison of Genotypes in T2DM Patients with Different TSH Levels

The distribution of the genotypes for FTO rs8050136 and rs9939609 conformed to HWE (*P* > 0.05), suggesting that these samples were representative of the population. The distribution of these alleles and genotypes was not statistically and significantly different between the two TSH groups (*P* > 0.05). When we applied the recessive model, both rs8050136 and rs9939609 were shown to be influencing factors for the TSH level (*P* < 0.05, as shown in Table 2). After adjusting for age, sex, and BMI, rs8050136 CC+CA genotype carriers were shown to express TSH that was 2.281 times more than their AA genotype counterparts (OR = 2.281, 95% CI = 1.024~5.080, *P* = 0.044), and rs9939609 TT+TA genotype carriers were shown to express TSH that was 2.417 times more than their AA genotype counterparts (OR = 2.417, 95% CI = 1.257-4.649, *P* = 0.008, as shown in Table 3).

The frequency of genotypes/alleles is presented as a percentage.

### 3.3. Comparison of TSH, FPG, and HbA1c Levels in Patients with Different rs8050136 and rs9939609 FTO Genotypes

**Figure 1:**
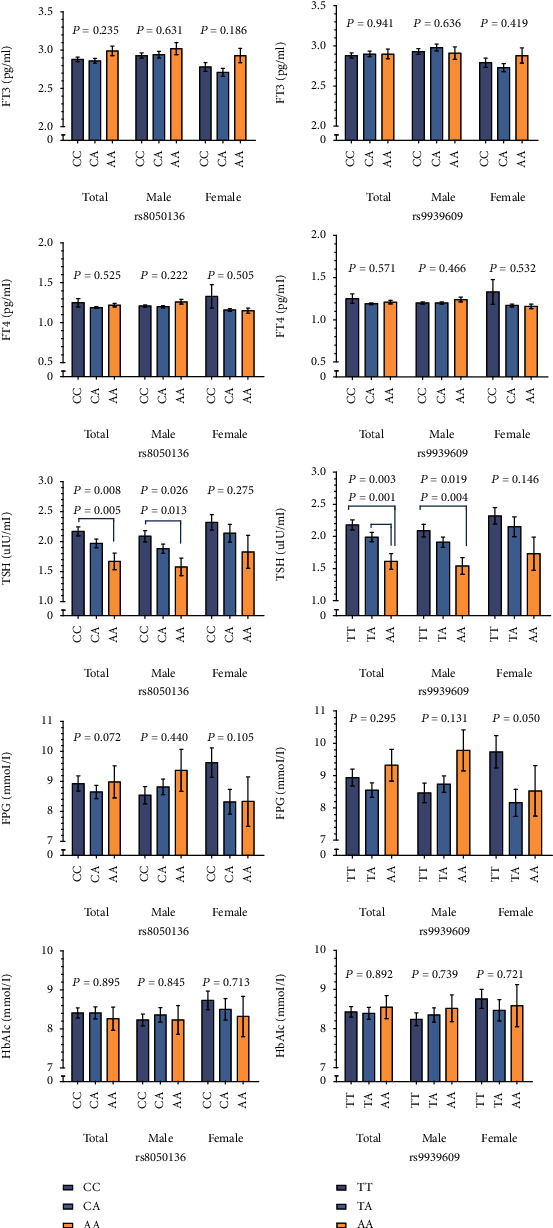
Comparison of TSH, FPG, and HbA1c levels in study participants with different FTO rs8050136 and rs9939609 genotypes.

TSH levels were different in T2DM patients with different FTO rs8050136 (*P* = 0.008) genotypes. The CC genotype carriers (2.17 ± 1.16) demonstrated more TSH than their AA genotype counterparts (1.67 ± 0.93, *P* = 0.005). Further analyzing TSH levels in different gender populations with different FTO rs8050136 genotypes, we found that the TSH levels were statistical different in male (*P* = 0.026). Intercomparison of three groups showed that the TSH levels of CC genotype (2.09 ± 1.15) were higher than those of AA genotype (1.58 ± 0.79, *P* = 0.013). TSH levels were also different in rs9939609 genotypes (*P* = 0.003), with TT (2.18 ± 1.16, *P* = 0.001) and TA (1.99 ± 1.05, *P* = 0.031) genotype carriers expressing significantly more TSH than their AA genotype counterparts (1.61 ± 0.90). The TSH levels of male were different among different FTO rs9939609 genotypes (*P* = 0.019), and the TT genotype carriers (2.09 ± 1.16) demonstrated more TSH than their AA genotype counterparts (1.54 ± 0.75, *P* = 0.004). Despite the differences in TSH, there were no significant differences in the FT3, FT4, FPG, and HbA1c levels between different rs8050136 and rs9939609 genotypes in T2DM patients (*P* > 0.05, as shown in Figure 1).

## 4. Discussion

The rapidly increasing prevalence of diabetes that affects blood vessels and nerves and accelerates the occurrence and development of neuropathy, microvascular, and macrovascular complications, as well as premature death, is a global public health problem. Developing countries, especially China, with a population of 116.4 million adults living with diabetes and with the highest prevalence of the disease in the world, are particularly at risk [1].

Studies have shown that the prevalence of thyroid dysfunction including both subclinical and significant hypothyroidism [6] is significantly increased in patients with T2DM [7, 8]. Changes in thyroid hormone levels in T2DM patients are associated with inflammation, cardiovascular events (CVE) [9, 10], peripheral neuropathy [11], diabetic nephropathy (DN) [12], and increased mortality. Even when the TSH levels are reported within the reference range, significant changes in these levels can predict fatal coronary heart disease. Changes in thyroid function are related to blood glucose, blood lipid, and IR in patients with diabetes, which may affect glucose tolerance and metabolic control and may have adverse effects on glucose control in patients with diabetes [13]. Patients with diabetes along with thyroid disease may be at increased risk for cardiovascular and microvascular complications [14]. Diabetes combined with hypothyroidism may damage vascular endothelium by inducing abnormal lipid metabolism, by increasing hypersensitivity, and by aggravating disorders in the blood clotting system. Underproduction of thyroid hormones may also affect the heart and cause myocardial damage, thereby increasing the risk of cardiovascular events. When the heart's ejection volume decreases, it might indirectly result in renal perfusion insufficiency and decreased glomerular filtration rates, thereby increasing the risk of nephropathy in these patients. Therefore, identifying the causes of thyroid dysfunction in T2DM patients and facilitating earlier assessment and effective intervention for these risk factors are the keys to the prevention and control of diabetes and its complications.

Genetic factors may be the basis underlying concurrent or successive onset of diabetes and thyroid disease. T2DM may also be associated with thyroid disease via genetic defects and susceptibility, as well as environmental, emotional, viral, and other factors. Studies have reported that HLA-DR3, CTLA4, and PTPN22 may be common genetic susceptibility genes for diabetes and hypothyroidism. The prevalence of T2DM is different between different populations. There are also differences in the impact on the Han and non-Han groups [15], especially in ethnic minority areas, like Xinjiang. The prevalence of T2DM in the Uyghur population in Xinjiang is significantly higher than that in the Han population as a result of genetic and lifestyle differences, including high BMIs, unhealthy diets, and low physical activity, which means that this group is also at a higher risk for the cardiovascular complications associated with diabetes [16]. Therefore, it is of great significance to select Uyghur population. FTO is located on chromosome 16, and SNPs in this locus have been linked to changes in fat mass and obesity, which are closely associated with type 2 diabetes [17]. Xiao et al. [18] reported that single-nucleotide mutations in rs8050136 and rs9939609 may be related to T2DM and obesity in Uyghur populations (*P* < 0.05). A study on children found a positive correlation between FTO rs9939609 polymorphism and serum TSH level (*β* = 0.10*Z*-score, *P* = 5.8 × 10^−3^) [5]. Therefore, the influence of FTO gene on the TSH level in T2DM patients in Xinjiang Uygur population was investigated.

In this study, 498 Uyghur T2DM patients were selected, 354 subjects with a TSH < 2.5 mU/L and 144 ones with a TSH ≥ 2.5 mU/L. The relationship between the TSH level and two common SNPs of FTO gene was studied in the subjects. We found that the recessive models of FTO SNPs rs8050136 (*P* = 0.047) and rs9939609 (*P* = 0.023) correlated with the TSH level in T2DM Uyghur patients in Xinjiang. The TSH level was also associated with the recessive models of rs8050136 (OR = 2.281, 95% CI = 1.024~5.080, *P* = 0.044) and rs9939609 (OR = 2.417, 95% CI = 1.257~4.649, *P* = 0.008) after adjusting for age, sex, and BMI. To our knowledge, this is the first study to demonstrate that variations in the FTO gene correlate with the TSH level in T2DM patients. Variations in FTO have been linked to weight gain and increased energy intake. Several studies on animal models have also shown that variations in FTO influence metabolic rates and energy expenditure [19, 20]. Additionally, FTO has been shown to exert an independent effect on BMI [21]. Genome-Wide Association Studies (GWAS) detected that common SNPs including rs8050136 and rs9939609 in the FTO gene were strongly associated with fat mass and obesity as well as increased risk for T2DM and thyroid disease [22, 23]. Although the association between FTO and obesity and diabetes mellitus has been demonstrated by many studies, the effects of FTO variation on TSH have not been reported. There was only one previous research that examined the relationship between FTO variation and childhood obesity which was conducted in India and found that rs9939609 variation was associated with the TSH level (*β* = 0.10*Z*-score, *P* = 5.8 × 10^−3^) and that mutations at rs8050136, which is in strong linkage disequilibrium with rs9939609 (*r*^2^ = 0.97), had similar results [5]. FTO is also widely expressed in the pituitary, and variations in FTO have been linked to changes in TSH production within these tissues. Studies have also shown that slight changes in TSH levels are associated with weight gain and increased fat mass [24]. However, the association between FTO alleles and TSH level and its BMI dependence on leptin levels suggests that FTO may be linked to metabolic regulation via the hypothalamic-pituitary-thyroid axis. And it is possible that FTO may play a role in the regulation of metabolism, possibly by regulating the expression of genes in metabolically active tissues [25].

We found that the TSH levels were significantly distinct in different rs8050136 and rs9939609 FTO genotypes. It suggests that genetic factors that influence obesity and T2DM may also play a role in the relationship between FTO and thyroid dysfunction in our study population. The thyroid gland is a critical component of the human endocrine system and continuous hyperglycemia, and the body's constant correction of carbohydrate imbalances has significant effects on thyroid activities [26]. Thyroid hormones stimulate glycogen synthesis and gluconeogenesis in the liver, thus aiding insulin in the regulation of the glucose metabolism. Thyroid hormones can also facilitate liver glucose release which may be regulated by the interactions of glucose transporter 4, insulin, and the hypothalamic [27]. Additionally, thyroid hormones also play an important regulatory role in insulin secretion and clearance. There was a study that had shown [28] that thyroid hormones regulate the behavior of many metabolic pathways associated with resting energy expenditure. TSH levels were positively correlated with BMI in obese patients with normal thyroid function. FTO is associated with fat mass and obesity, while TSH, a crucial hormone regulating energy metabolism, can affect lipid metabolism via a thyroid-independent pathway. However, there was no statistical significance between FTO and other metabolic indices such as the levels of FT3, FT4, FPG, and HbA1c. Two studies reported that the FTO polymorphism rs9939609 was not associated with levels of fasting glucose [29, 30]. The observed results might be due to insufficient statistical power contributed by small sample size. It may also be related to the limitations of the study population we selected.

There was a difference in metabolic indexes between male and female due to hormonal differences. Therefore, we further compared the relationship between different genotypes of rs8050136 and rs9939609 of FTO gene and TSH level and other metabolic indicators in different gender population. The significant association between rs8050136 and rs9939609 genotypes of FTO and TSH levels was found only in male patients. It indicated that the TSH levels related to the genotypes of rs8050136 and rs9939609 of FTO gene in the male population were different from those in the female population. The reason and whether other loci of FTO gene also have same relationship with TSH levels remains to be further studied.

There are some limitations in this study. First, the sample size of our study is relatively small, reducing the ability of genetic association, and there may be insufficient statistical power to detect weak genetic effects, resulting in fluctuations in estimates. Secondly, we assessed the correlation between FTO gene polymorphism and TSH level in diabetic patients, and there may be other unstudied SNPS affecting the TSH level. Third, our study population is the Uyghur, which is a mixed population originated from intermarriage between Caucasians and Mongolians. The Uygur is also a homogenous population which have their own genetic background, language, culture, lifestyle, and dietary habits. This special genetic background affects the strength and direction of genetic association.

## 5. Conclusions

We found that the polymorphism of FTO genes rs8050136 and rs9939609 was correlated with the serum TSH level in T2DM patients in Xinjiang Uyghur population. But the difference was only found in male T2DM patients. Patients especially men with diabetes should be screened for related risk factors and implemented intervention of mental health early. Thyroid function should also be regularly investigated based on the complex relationship between diabetes and thyroid dysfunction, as well as the role of FTO gene polymorphism in diabetes and thyroid dysfunction. In addition, it is necessary to further explore the influence of FTO gene polymorphisms rs8050136 and rs9939609 on the TSH level and the possible mechanism in different populations.

## Figures and Tables

**Table 1:** Summary of the general clinical information available for each of the study participants including their TSH level.

Group	*n*	Male	Female	Age (years)	BMI	SBP	DBP	FPG
TSH-0	354	241 (68.08%)	113 (31.92%)	51.03 ± 9.52	27.80 ± 3.81	128.20 ± 17.23	80.16 ± 11.09	9.05 ± 3.88
TSH-1	144	83 (57.64%)	61 (42.36%)	51.69 ± 9.29	28.66 ± 4.39	127.89 ± 18.78	79.71 ± 12.26	8.36 ± 3.29
Total	498	324 (65.06%)	174 (34.94%)	51.22 ± 9.45	28.05 ± 4.00	128.11 ± 17.68	80.03 ± 11.43	8.85 ± 3.73
*χ* ^2^ (F)		4.908		0.500	4.775	0.032	0.154	3.492
*P*		0.027		0.480	0.029	0.858	0.695	0.062

Group	n	HbA1c	FT3	FT4	TC	TG	HDL-C	LDL-C
TSH-0	354	8.48 ± 2.15	2.87 ± 0.46	1.20 ± 0.17	4.48 ± 1.12	2.37 ± 1.97	0.92 ± 0.28	2.72 ± 0.87
TSH-1	144	8.21 ± 2.00	2.92 ± 0.49	1.27 ± 1.00	4.62 ± 1.18	2.31 ± 1.89	0.97 ± 0.30	2.87 ± 0.87
Total	498	8.40 ± 2.11	2.88 ± 0.47	1.22 ± 0.56	4.52 ± 1.13	2.35 ± 1.95	0.93 ± 0.28	2.76 ± 0.87
*χ* ^2^ (F)		1.664	1.257	1.206	1.485	0.091	3.798	2.995
*P*		0.198	0.209	0.228	0.224	0.763	0.052	0.084

**Table 2:** Association between FTO rs8050136 and rs9939609 and the TSH level in study participants.

SNP	Group	Allele		Genotype			Dominant model		Recessive model	
		C	A	CC	CA	AA	CC	CA+AA	CC+CA	AA
rs8050136	TSH-0 (*n* = 343)	460 (67.06)	226 (32.94)	156 (45.48)	148 (43.15)	39 (11.37)	156 (45.48)	187 (54.52)	304 (88.63)	39 (11.37)
	TSH-1 (*n* = 144)	210 (72.92)	78 (27.08)	74 (51.39)	62 (43.06)	8 (5.56)	74 (51.39)	70 (48.62)	136 (94.45)	8 (5.56)
	*χ* ^2^	3.246		4.303			1.42		3.933	
	*P*	0.720		0.116			0.233		0.047	

	Group	Allele		Genotype			Dominant model		Recessive model	
		T	A	TT	TA	AA	TT	TA+AA	TT+TA	AA
rs9939609	TSH-0 (*n* = 340)	447 (65.74)	233 (34.26)	151 (44.41)	145 (42.65)	44 (12.94)	151 (44.41)	189 (55.59)	296 (87.06)	44 (12.94)
	TSH-1 (*n* = 138)	198 (71.74)	78 (28.26)	68 (49.28)	62 (44.92)	8 (5.80)	68 (49.28)	70 (50.72)	130 (94.20)	8 (5.80)
	*χ* ^2^	3.224		5.23			0.935		5.167	
	*P*	0.073		0.073			0.333		0.023	

**Table 3:** The results of the logistic regression analysis between FTO rs8050136 and rs9939609 and TSH level in study participants when evaluated using the recessive model.

SNP	Genetic model	TSH-0/TSH-1 (*n*)	OR (95% CI)	*P*	Adjusted OR (95% CI^∗^)	Adjusted *P*^∗^
rs8050136	Recessive model					
	AA	39/8	1		1	
	CC+CA	304/136	2.181 (0.993, 4.792)	0.052	2.281 (1.024, 5.080)	0.044

rs9939609	Recessive model					
	AA	44/8	1		1	
	TT+TA	296/130	2.311 (1.210, 4.411)	0.011	2.417 (1.257, 4.649)	0.008

^∗^Adjusted for age, sex, and BMI. OR: odds ratio; 95% CI: 95% confidence interval.

## Data Availability

The analyzed data used to support the findings of this study are available from the corresponding author on reasonable request.
